# A study on the influence of academic passion on PhD students’ research engagement—The role of ambidextrous learning and academic climate

**DOI:** 10.1371/journal.pone.0303275

**Published:** 2024-06-03

**Authors:** Jianyue Chen, Zhixing Zhao

**Affiliations:** 1 School of Public Policy and Management, China University of Mining and Technology, Xuzhou, China; 2 Sichuan Institute of Higher Studies in Culture and Education, Sichuan Normal University, Chengdu, China; Universiti Pertahanan Nasional Malaysia, MALAYSIA

## Abstract

The engagement in research, as the primary form of learning engagement for PhD students, is crucial for enhancing their competitive edge. Academic passion, a key determinant of learning engagement, plays a significant role in driving the research enthusiasm of PhD students. However, the "black box" concerning whether and how academic passion influences PhD students’ research engagement remains to be explored. Addressing this gap, the present study draws upon self-determination theory, adopts the "motivation-behavior-effect" analytical framework, and incorporates ambidextrous learning as a mediator to elucidate the specific pathway through which academic passion impacts PhD students’ engagement in research activities. Furthermore, it examines the facilitating role of the academic climate in this process. From December 2022 to March 2023, a questionnaire survey was conducted, collecting 522 responses from PhD students across 25 universities in China. The survey primarily assessed the PhD students’ academic passion, ambidextrous learning behaviors (including tendencies towards exploratory and exploitative learning), and their perceived academic climate, investigating how these factors collectively influence their engagement in research activities. The questionnaire data were analyzed using a combination of SEM and bootstrapping with SPSS 26.0 and Mplus 8.3 software. The findings reveal that academic passion significantly positively affects PhD students’ research engagement; ambidextrous learning (exploratory and exploitative learning) mediates the relationship between academic passion and PhD students’ research engagement; and the academic climate effectively facilitates the transformation of PhD students’ academic passion into ambidextrous learning (exploratory and exploitative learning). The study’s conclusions not only foster PhD students’ enthusiasm for research but also enhance learning effectiveness and innovation vitality, providing a theoretical basis for reforming the doctoral training system.

## 1. Introduction

In the context of globalization and the knowledge economy, doctoral education plays an indispensable role in national efforts to reserve high-level human capital and seize the forefront of scientific and technological advancement [1]. It contributes significantly to societal progress and technological development. Research engagement, characterized by the depth of involvement and sustained effort PhD students exhibit in scientific exploration, is a decisive factor affecting their academic achievements and personal development [2, 3], as well as a key pathway to cultivate their innovative thinking and capabilities [4]. The intensity and quality of research engagement directly determine whether PhD students can stand out in the competitive academic field, pioneer new knowledge domains, and achieve breakthroughs in research. Deepening our understanding of this process, particularly the intrinsic mechanisms that stimulate and sustain PhD students’ research engagement, is crucial for personal growth and the enhancement of scientific innovation capabilities.

Self-Determination Theory (SDT) offers a powerful framework for understanding individual intrinsic motivation, emphasizing that the satisfaction of autonomy, competence, and relatedness is key to spurring individuals’ positivity [5]. Building on this theory, academic passion is seen as a tendency of researchers to invest time and energy in research activities [6], serving not only as an intrinsic motivation driving graduate students towards degree attainment [7] but also as an important force in achieving academic goals [8]. Although existing research has paid some attention to academic passion and its role in promoting learning and research, particularly among high school and undergraduate populations [9, 10], studies focusing on the unique group of doctoral students, especially on how academic passion influences research engagement through specific learning mechanisms, are relatively scarce.

Enhancing research engagement relies not only on the effective use of existing knowledge resources but also on the continuous exploration of new knowledge. This dual pursuit of knowledge, known as ambidextrous learning, where exploratory learning focuses on the discovery and innovation of new resources [11], and exploitative learning emphasizes the integration and application of existing resources [12], has become a critical aspect of research activities. The adoption of ambidextrous learning strategies has stimulated innovative thinking among PhD students while maintaining knowledge updates. However, despite the growing recognition of the importance of ambidextrous learning in enhancing research engagement and innovation efficiency, how academic passion influences PhD students’ research engagement through the framework of ambidextrous learning, and how the academic climate facilitates this process remains underexplored.SDT suggests that individual behavior results from the interplay between intrinsic motivation and external environmental factors, providing a theoretical basis for understanding the relationships among ambidextrous learning, academic passion, and research engagement [13, 14]. The academic climate, as a key organizational environmental factor in higher education institutions, significantly impacts PhD students’ academic outcomes and innovative capabilities [15, 16]. Current research lacks in-depth discussion on how academic passion influences PhD students’ research engagement through ambidextrous learning. Additionally, how the academic climate as an external factor affects this dynamic interaction process has not been sufficiently addressed. Therefore, this study aims to fill this gap by exploring the mediating role of ambidextrous learning in the relationship between academic passion and research engagement, and how the academic climate moderates this relationship, offering new perspectives for understanding and promoting PhD students’ research engagement.

## 2. Literature review and research hypothesis

### 2.1. Academic passion and research engagement

Self-Determination Theory (SDT), proposed by Deci and Ryan in the early 1980s, aims to explore how individuals autonomously determine and regulate their behavioral motivations. SDT is founded on a core premise that humans are inherently proactive beings with an intrinsic drive for growth and development, stemming from the fulfillment of three basic psychological needs: autonomy, competence, and relatedness. Autonomy refers to the capacity for self-determination and personal choice in one’s actions; competence involves an individual’s perception of their ability to impact their environment and achieve goals; relatedness denotes the need to form meaningful connections with others [17]. SDT provides robust theoretical support and explanatory power for understanding and promoting positive behaviors in education, work, and other life domains. In education, SDT emphasizes the importance of providing a learning environment that supports students’ autonomy, competence, and relatedness to foster intrinsic learning motivation, enhance learning efficiency, and academic achievement [18, 19]. In organizational management, SDT is utilized to explain employee satisfaction, job engagement, and organizational loyalty [20–22]. SDT is also applied in personal life and health promotion, particularly in explaining how people engage in health behavior changes [23]. In summary, Self-Determination Theory (SDT) becomes the theoretical foundation of this study due to its deep exploration of individual behavioral motivations and its potential in elucidating the mechanisms through which academic passion affects PhD students’ engagement in scientific research. The theory not only clarifies how intrinsic motivation can inspire doctoral students to continuously engage in scientific activities but also guides how to create a supportive academic environment to foster and maintain research engagement, ensuring the appropriateness of theoretical selection and clarity of practical guidance for the study.

Psychologist Vallerand [24] defines passion as an individual’s willingness to invest significant time and energy in an activity, manifesting as a strong inclination towards identification, liking, or even love. Passion predicts higher levels of vitality and a sense of efficacy, motivating individuals to invest more time and resources, and to persevere more intensely [25]. Academic passion, the application of passion in the academic domain, is the time and energy students invest in academic exchanges, research projects, and writing [6]. Academic passion positively predicts students’ academic functioning, affecting learning engagement and resulting in positive academic outcomes [26]. Existing research has mostly focused on the impact of academic passion on academic outcomes and its antecedents [27, 28], overlooking the consideration of psychological need satisfaction on doctoral students’ research engagement. Therefore, applying SDT in this study not only fills the research gap on the mechanism of influence of doctoral students’ academic passion on research engagement but also provides a novel perspective to understand and promote sustained participation of doctoral students in scientific activities.

Existing studies indicate that learning engagement, as the level of effort and the sustained and positive emotional state students display in participating in learning activities and solving learning problems [29], reflects students’ learning capabilities and is an important indicator for observing the learning process and assessing learning quality [30, 31]. Research engagement, as the core of doctoral students’ academic journey, reflect their passion, dedication, and focus on scientific exploration, which interact and influence each other in the research exploration [32]. Self-Determination Theory (SDT) provides a perspective on how individuals can ignite passion, behavior, and performance through the process of internalizing specific activities into their identity [33]. Studies show that high levels of passion can evoke more positive emotions in individuals, compelling them to continue investing cognitive resources, time, and energy [34], thereby significantly enhancing the state of engagement [35, 36]. Particularly, when doctoral students can internalize academic research as part of their identity, they are more likely to ignite a strong academic passion. This passion keeps doctoral students highly interested and positively emotionally engaged in research activities, enables them to bravely face complex challenges in research, and persevere through difficulties, thus investing more effort into their scientific endeavors.In sum, we propose the following:

**Hypothesis 1: Academic passion has a significant positive effect on PhD students’ research engagement**

### 2.2. The mediating effect of ambidextrous learning

The transformation of academic passion into research engagement essentially embodies an innovation process [37], where innovation primarily stems from the fusion of various technologies and types of knowledge. Through learning, individuals can acquire, share, transform, and apply beneficial knowledge to achieve set goals [38]. Self-Determination Theory (SDT) highlights how both intrinsic and extrinsic motivations influence the selection, intensity, and persistence of individual behaviors [39]. Within this theoretical lens, ambidextrous learning behaviors provide a framework for understanding how doctoral students, influenced by motivational factors in SDT, impact their research engagement through diverse learning paths. Ambidextrous learning, entailing both exploratory and exploitative learning modes, not only emphasizes the importance of balancing innovation with maintaining existing achievements but also illustrates how organizations and individuals can acquire knowledge through this dual pathway [40]. The distinction between exploratory and exploitative learning lies in their respective focuses on exploring new knowledge and applying existing knowledge. Exploratory learning concentrates on the discovery, experimentation, and risk-taking associated with new knowledge, aiming for innovation and adaptability to change; conversely, exploitative learning focuses on deepening and optimizing existing knowledge and skills, aiming to enhance efficiency and quality of outcomes. The combination of these dual learning strategies not only meets individuals’ needs for innovation but also ensures the stability and reliability of outcomes [41].

In the research activities of doctoral students, intrinsic motivations (such as a love for research, self-fulfillment, and a sense of achievement) inspire their curiosity for new knowledge and pursuit of innovative solutions to research problems [42]. At the heart of exploratory learning is doctoral students’ in-depth exploration of previously untouched areas and acquisition of knowledge and skills, continually pushing them beyond existing knowledge boundaries and opening new channels of understanding, thereby enhancing their innovation capability and performance [43]. Although this learning approach may bring significant knowledge gains, it also comes with unpredictable risks [44], requiring doctoral students to possess high professional skills and knowledge reserves and to invest more effort. Here, academic passion becomes a key driving force, motivating doctoral students to fully engage in research exploration, persist through difficulties and challenges, and demonstrate a high level of commitment necessary for achieving excellence [45]. Thus, influenced by academic passion, exploratory learning not only effectively enhances doctoral students’ research engagement but also lays a solid foundation for their research innovation.

Extrinsic motivations (such as academic and career advancement, and rewards and recognition from the academic community) also play a crucial role in the research activities of doctoral students [46], potentially motivating them to efficiently use existing resources and optimize research methods to achieve research goals. Exploitative learning focuses on stimulating innovative insights by deeply mining and refining existing knowledge resources [47], enabling doctoral students to perfect and integrate mastered knowledge and skills, thereby expanding their cognitive breadth and depth. This learning mode not only enhances individual learning capacity [48] but also helps teams maintain competitive advantages and create new value [49], especially beneficial for enhancing short-term research performance due to its clarity and proximity [50].Self-Determination Theory indicates that intrinsic and extrinsic motivations can interact and transform, with intrinsic motivation being more conducive to stimulating learning behavior [51], increasing personal vitality [52], and investment in learning [53]. Thus, in research activities, when extrinsic incentives align with doctoral students’ values and interests, extrinsic motivation can be internalized, becoming an intrinsic driving force to enhance research engagement. This internalization process promotes deeper and sustained involvement of doctoral students in research activities, increasing their satisfaction with and the quality of engagement in the research process.

In summary, academic passion is not only an intrinsic driving force propelling doctoral students into research but also a key factor in their choice between exploratory and exploitative learning paths. Doctoral students with high academic passion tend to favor exploratory learning, daring to venture into unknown territories in pursuit of new knowledge and innovative methods. This learning approach encourages them to challenge existing boundaries, fostering an enhancement of innovation capability and academic achievement. Conversely, those who maintain a steady passion for specific research areas may prefer exploitative learning, seeking to improve research efficiency and quality by deepening and optimizing existing knowledge and skills. These tendencies not only reflect doctoral students’ personal preferences for research activities but are also influenced by the academic environment and specific research tasks. Therefore, ambidextrous learning behaviors provide an effective framework for understanding how academic passion fosters doctoral students’ research engagement. By balancing the impulse to explore new fields with the need to optimize existing outcomes, doctoral students can find an appropriate equilibrium between research innovation and outcome stability, crucial for both their research output generation and personal career development.In light of this, we suggest the following:

**Hypothesis 2: Exploratory learning mediates the relationship between academic passion and PhD students’ research engagement**
**Hypothesis 3: Exploitative learning mediates the relationship between academic passion and PhD students’ research engagement**

### 2.3. The moderating role of the academic climate

The complexity of individual learning processes stems not only from the diversity of intrinsic motivations and individual differences but also from the significant influence of the external environment [54]. Self-Determination Theory (SDT) emphasizes that the external environment drives effective behaviors by fostering internal motivations and the internalization of external motivations [5], with the manner of individual internalization depending on specific situational elements. Thus, SDT provides a robust framework for understanding and analyzing how individuals are influenced by the external environment, particularly in exploring how the academic climate modulates the relationship between academic passion and doctoral students’ research engagement.

Existing research indicates that the atmosphere, as a crucial factor affecting students’ learning activities [55], facilitates the regulation of student emotions [56] and enhances learning outcomes. Different types of atmospheres exist in various contexts, with the academic atmosphere being the essence and deep cultural accumulation formed by various academic activities within universities, subtly influencing the thoughts and psychological perceptions of members within the institution [57]. Its intensity determines the academic behaviors and research outcomes of academic members within the institution [58]. However, most of these studies focus on the general learning environment [59, 60], with fewer explorations into the impact of academic atmosphere on the specific group of doctoral students. As the main force in academic research, doctoral students’ research engagement is driven not only by their intrinsic academic passion but also significantly influenced by the surrounding academic environment—especially the academic climate.

Specifically, when schools and teams provide as much resource support and learning exchange opportunities as possible, placing doctoral students in a "strong situation" with a rich academic atmosphere, these students, influenced by the academic climate, tend to actively engage in communication and learning exchanges with other members [61]. They attempt to integrate and utilize knowledge, enhance learning efficiency to complete tasks, and gain recognition from others. Conversely, in a poor academic atmosphere, where doctoral students are in a "weak situation, " it may negatively guide their psychology, behavior, and thoughts [62], leading to a decline in academic passion and a lack of enthusiasm for research and innovation.Therefore, we argue the following:

**Hypothesis 4: The academic climate positively moderates the direct relationship between academic passion and exploratory learning, and the higher the academic climate, the stronger the relationship.**
**Hypothesis 5: The academic climate positively moderates the direct relationship between academic passion and exploitative learning, and the higher the academic climate, the stronger the relationship.**

In summary, the academic climate modulates the indirect relationship between academic passion and doctoral students’ research engagement, with ambidextrous learning acting as the mediating variable. When doctoral students are in a positive and supportive academic atmosphere, they are more likely to be positively influenced by mentor guidance and peer interaction, thereby stimulating academic passion and promoting the development of exploratory and exploitative learning behaviors. This favorable academic atmosphere not only strengthens the positive effects of academic passion on ambidextrous learning but also, by fostering more effective learning behaviors, further enhances doctoral students’ research engagement. Conversely, in environments where the academic atmosphere is weaker, doctoral students may face a lack of sufficient mentor support and peer interaction. This can lead to inadequate stimulation of academic passion, thereby inhibiting the emergence of learning behaviors and affecting the effectiveness of their research engagement. The following theories are put out in this study in light of this:

**Hypothesis 6: Academic climate positively moderates the indirect effect of academic passion on PhD students’ research engagement through exploratory learning, and the higher the academic climate, the stronger the relationship.**
**Hypothesis 7: Academic climate positively moderates the indirect effect of academic passion on PhD students’ research engagement through exploitative learning, and the higher the academic climate, the stronger the relationship.**

**Fig 1:**
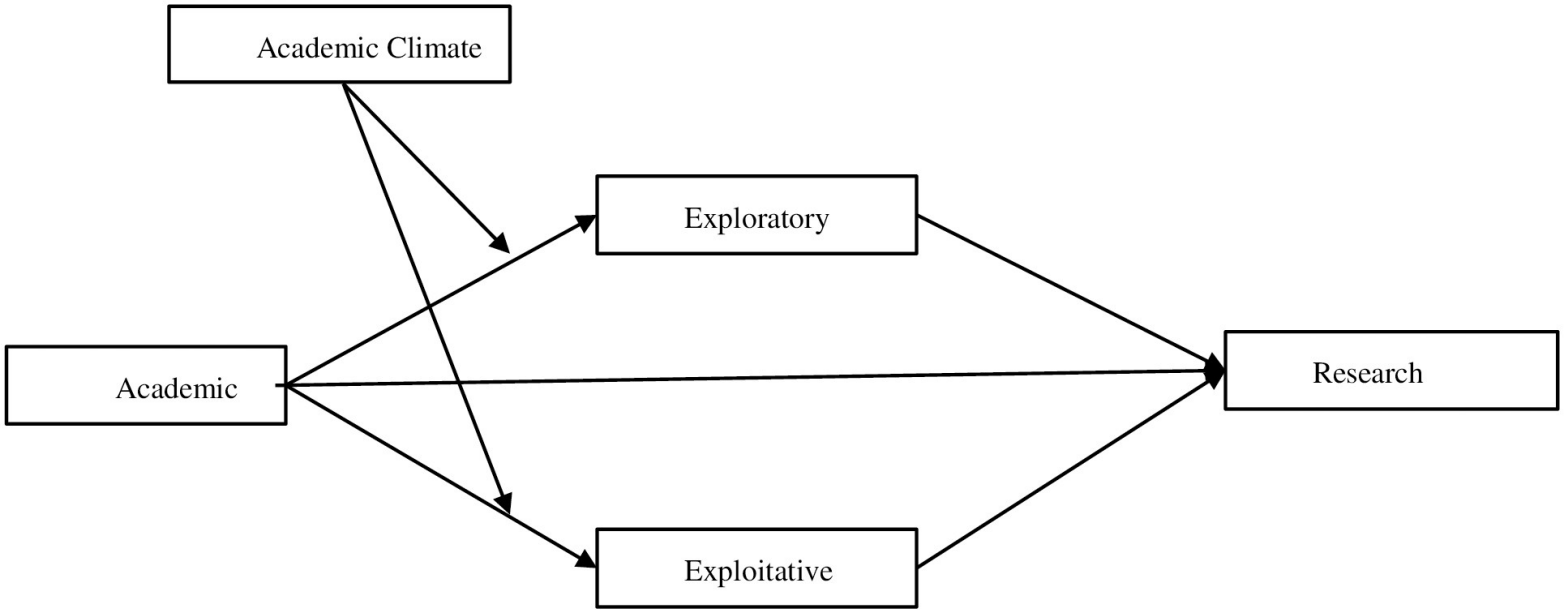
Research model.

This study constructs a theoretical conceptual model as shown in Fig 1.

## 3. Method

### 3.1. Sample and procedure

This study strictly adhered to the standards and guidelines of ethical research and received approval from the Ethics Committee of the School of Public Policy and Management, China University of Mining and Technology (Approval number: 2022BL-010-05). The research sample was selected from doctoral students currently enrolled at 25 universities in provinces such as Hubei, Jiangsu, Sichuan, and Shaanxi. The distribution of the sample is shown in Table 1. To obtain a larger number of valid samples, some surveys were conducted with the assistance of university admissions departments via the distribution of electronic questionnaires. To alleviate respondents’ concerns and enhance the validity of the questionnaires, this study adopted anonymous and sensitive wording in the questionnaire design. Before the commencement of data collection, all potential participants were clearly informed about the purpose and intent of the research, and a "Participant Information and Consent Form" was provided to the respondents. This form detailed the privacy and data protection measures for participants, as well as their right to withdraw from the study at any time, ensuring voluntary participation and transparency of information.To reduce common method bias, the data collection lasted for three months from December 2022 to March 2023, and three waves of data collection were selected at different time points. Time point 1: The scales of academic passion, research engagement and control variables of PhD students were collected, 1500 questionnaires were distributed, 1045 data were collected, 856 questionnaires were valid, and the first effective recovery rate was 81.914%;Time point 2: After four weeks, the 856 valid questionnaires collected at time point 1 were followed up and the scale for ambidextrous learning was collected and 672 data were retrieved, of which 641 were valid, with a second validity rate of 95.387%; Time point 3: After four weeks, the 641 valid questionnaires collected at time point 2 were followed up with a scale of academic climate, 526 data were collected, 522 valid data were collected and the validity rate for the third time was 99.24%.

**Table 1:** Sample distribution.

Title	Category	Sample size	Percentage	Title	Category	Sample size	Percentage
Population Statistics	Male	360	69%	School Type	Double first-rate universities	357	68.4%
	Female	162	31%		General university	165	31.6%
Grade Composition	Year1	188	36%	Admission Method	Master’s direct doctoral degree	247	47.3%
	Year2	146	28%		Application for review	63	12.1%
	Year3	106	20.3%		Common examination	212	40.6%
	Year4	55	10.5%	Subject Classification	Natural sciences	226	43.3%
	Year5+	27	5.2%		Human sciences	296	56.7%

### 3.2. Measures

To ensure the reliability of the measurement instrument, this study used a widely used and established scale from authoritative domestic and international journals for the measurement of the relevant variables. For the purpose and requirements of this study, the translation-back translation procedure is strictly followed, and appropriate optimization is made through expert discussion, striving for accurate semantic expressions, standardized presentation forms, and compliance with Chinese language standards. All scales were scored on a 7-point Likert scale, with "1–7" indicating the level of endorsement of the question item.

*Academic passion*. Using Stoeber et al. (2011) [6] adapted from the 2-dimensional 10-item scale developed by Vallerand (2008) [63], which is widely used to measure the degree of academic passion. Sample items included "Being in academia makes me feel fulfilled" and "My emotional well-being often depends on the progress of my academic activities". Cronbach’s α was 0.956.

*Exploratory learning*. Based on the Atuahene-Gima & Murray (2007) [64] scale, the scale was combined with the characteristics of individual PhD students’ learning behaviors, and was consolidated with appropriate additions and deletions. Sample items included "I learn to acquire new knowledge and conduct new research" and "I tend to use new methods to solve problems". Cronbach’s α was 0.908.

*Exploitative learning*. Based on the Atuahene-Gima & Murray (2007) [64] scale, the scale was combined with the characteristics of individual PhD students’ learning behaviors, and was consolidated with appropriate additions and deletions. Sample items included "I study to improve the efficiency and quality of my current research" and "I often use proven, generally accepted solutions to problems". Cronbach’s α was 0.911.

*Academic climate*. The scale developed by Wang et al.(2013) [65], consisting of six items, was utilized. This scale was designed based on the actual conditions of the academic environment in Chinese universities, aiming to comprehensively capture environmental factors affecting doctoral students’ academic growth, including the richness of academic exchange activities and the adequacy of academic resources. Example items include: “My school and team have a wealth of academic exchange activities” and “My school and team have ample academic resources”. Cronbach’s α was 0.931.

*Research engagement*. The 17-item three-dimensional scale developed by Schaufeli et al. (2002) [66] was used: 6 questions on the vitality dimension, 5 questions on the dedication dimension, and 6 questions on the concentration dimension. Sample items included "I’m happy to get up in the morning to do academic research" and " Academic research inspires me". Cronbach’s α was 0.975.

*Control variables*. Through a review of existing literature, it has been found that characteristics such as doctoral students’ personal backgrounds and academic environment have a significant impact on their research engagement. Specifically, age, gender, and academic year are key personal background factors affecting research engagement, while discipline type, academic level of the institution, and mode of admission reflect the academic environment and resource conditions of doctoral students [32, 67–69]. Notably, the size of the advisor’s research team plays an important role in supporting doctoral students’ research activities. The team size may affect doctoral students’ opportunities to receive guidance, access research resources, and collaboration opportunities, thereby impacting their research output and engagement [70, 71]. Therefore, this study considers gender, age, academic year, discipline type, academic level of the institution, mode of admission, and the size of the advisor’s research team as control variables in the research.

### 3.3. Analysis methods

SPSS 26.0 and Mplus 8.3 were used for statistical analysis in this study. First, the main variables were tested for homoscedasticity by Harman’s one-factor test, and validation factor analysis was conducted to test the inter-variate discriminant validity; Second, presenting descriptive statistics and correlation analysis between variables to initially test the main effects hypothesis; Third, the model main effects and mediating effects were tested using path coefficient plots and bootstrap methods; Finally, the moderating effect was tested against the conditional process model using SEM.

## 4. Results

### 4.1. Common source bias and confirmatory factor analysis

As the data collection was generated through self-assessment by PhD students, common source bias was difficult to avoid, although the design avoided it by specifying the purpose of the study, emphasizing confidentiality of information, and filling in responses at multiple waves. To ensure data rigor, the Harman one-factor test was used to test for common source bias, and the results revealed that the unrotated first factor explained 38.055% (less than 40%) of the variance, which tentatively suggests that the problem of common source bias is not serious. Further, with the aid of the single latent factor method, a common method latent factor is added to the structural equation model and the change in model fit after the addition of this latent variable is compared. The results show that the fit of the model to the data does not change significantly after controlling for the common method factor(Δχ^2^ / *df* = 0.402, ΔRMSEA = 0.007, ΔCFI = 0.003, ΔTLI = 0.007). Consequently, this study does not have a serious common source variance problem and subsequent data analysis can be performed.

A validated factor analysis was conducted using Mplus 8.3 on the main variables: academic passion, exploratory learning, exploitative learning, academic climate, and research engagement, to evaluate the inter-variate discriminant validity. According to the scale factor fit index in Table 2, the five-factor model fit was relatively good (χ2/*df* = 1.182, RMSEA = 0.019, CFI = 0.992, TLI = 0.991, SRMR = 0.024), which was significantly better than the other 4 alternative models, indicating good discriminant validity among the 5 main variables.

**Table 2:** Fitness indexes of scales.

	*χ* ^2^	*df*	RMSEA	CFI	TLI	SRMR
Model 1: Five factor model	1004.797	850	0.019	0.992	0.991	0.024
Model 2: Four-factor model	2416.291	854	0.059	0.918	0.913	0.068
Model 3: Three-factor model	4026.371	857	0.084	0.834	0.825	0.123
Model 4: Two-factor model	6313.869	859	0.110	0.714	0.700	0.151
Model 5: One-factor model	9441.549	860	0.138	0.550	0.528	0.156

Note.Model 2: "Exploratory Learning + Exploitative Learning" combined into one factor; Model 3: "Academic Passion + Exploratory Learning + Exploitative Learning" combined into one factor; Model 4: "Academic Passion + Exploratory Learning + Exploitative Learning + Academic Climate" combined into one factor; Model 5: All variables combined into one factor.

### 4.2. Descriptive statistics and correlation analysis

The variable means, standard deviations, and correlation coefficients between the variables are shown in Table 3. Academic passion with the mediating variables exploratory learning (*r* = 0.283, *p*<0.01), exploitative learning (*r* = 0.290, *p*<0.01), and the outcome variable research engagement (*r* = 0.406, *p*<0.01); Mediating variable exploratory learning and outcome variable research engagement (*r* = 0.420, *p*<0.01); Mediating variable exploitative learning and outcome variable research engagement (*r* = 0.473, *p*<0.01).Consequently, the results of the correlation analysis tentatively support the correlation hypothesis.

**Table 3:** Descriptive statistics and correlation coefficient of variables.

Variable	1	2	3	4	5	6	7	8	9	10	11	12
Gender												
Age	-0.045											
Grade	0.053	-0.025										
Subject	-0.075	0.002	0.018									
School type	-0.073	0.000	-0.040	0.055								
Admission Method	-0.032	0.038	-0.050	0.029	0.036							
Team size	0.053	-0.051	0.032	-0.049	-0.048	-0.020						
Academic Passion	0.042	-0.023	-0.053	-0.032	-0.078	-0.058	0.087*	**0.956**				
Exploratory Learning	-0.043	-0.085	-.115**	-0.001	-0.094*	0.037	0.107*	0.283**	**0.908**			
Exploitative Learning	0.029	-0.083	-0.027	-0.012	-0.089*	0.035	0.135**	0.290**	0.355**	**0.911**		
Academic Climate	-0.009	-0.055	-0.058	0.041	-0.053	0.045	0.088*	0.214**	0.294**	0.301**	**0.931**	
Research Engagement	0.082	-0.047	-0.012	-0.036	-.260**	-0.024	0.233**	0.406**	0.420**	0.473**	0.281**	**0.975**

Note. n = 522;

*p<0.05,

**p<0.01,

***p<0.001.

### 4.3. Hypotheses testing

**Fig 2:**
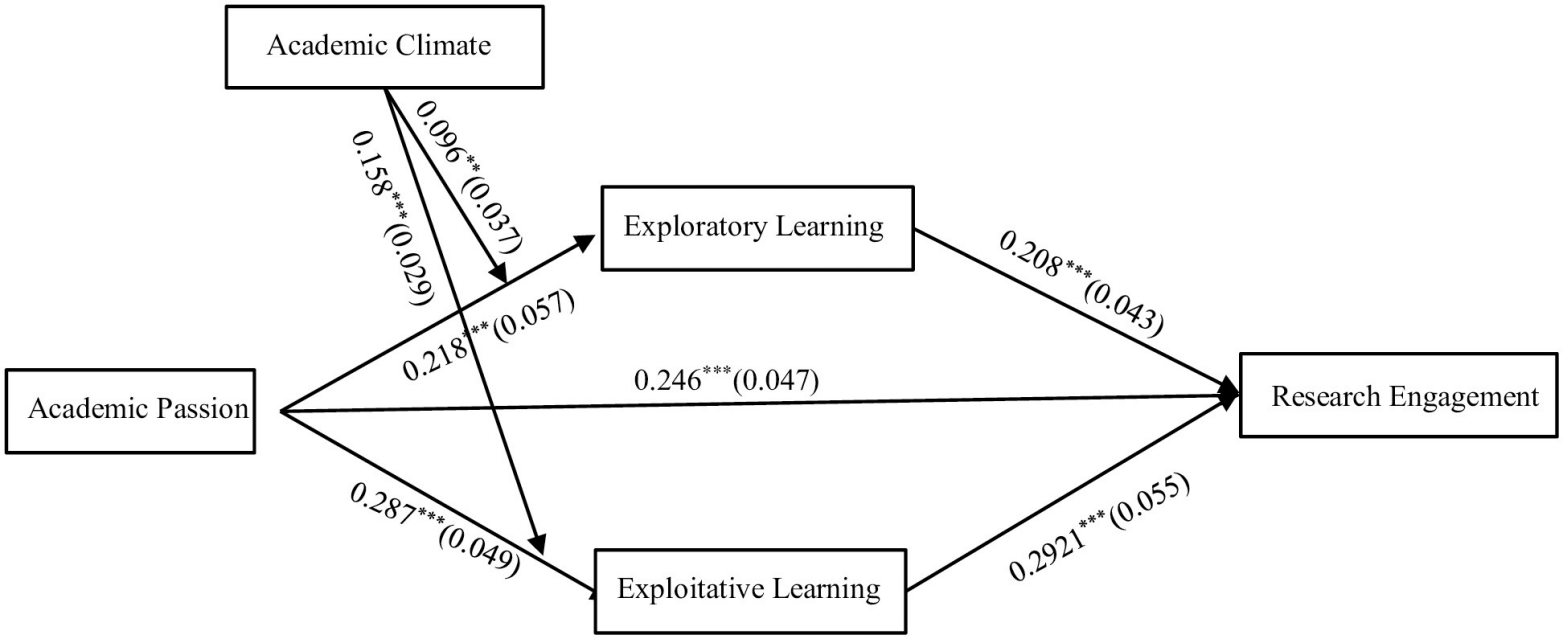
Path coefficient diagram.

*Main effect test*. In this study, the main effects were tested using the Mplus 8.3 full model, and the results of the full model test are shown in Fig 2, which shows that: Academic passion positively influenced exploratory learning (*r* = 0.218, *p*<0.001), exploitative learning(*r* = 0.287, *p*<0.049) and research engagement(*r* = 0.246, *p*<0.001), thus Hypothesis 1 is supported.

*Mediation effects test*. The results of the bootstrapping mediated effects test with a sample size of 10, 000 for the path coefficient analysis of the variables with the use of Mplus 8.3 showed that: The indirect effect value of academic passion through exploratory learning on research engagement was 0.045 with a 95% confidence interval of [0.018, 0.068]. The indirect effect value of academic passion on research engagement through exploitative learning was 0.060 with a 95% confidence interval of [0.033, 0.096], and Hypothesis 2 and Hypothesis 3 are supported.

*Moderation effect test*. As shown in Fig 2, the academic climate had a positive moderating effect between academic passion and exploratory learning (*r* = 0.096, *p*<0.001), and academic climate also had a positive moderating effect between academic passion and exploitative learning (*r* = 0.158, *p*<0.001). The moderating effect was initially validated.

**Fig 3:**
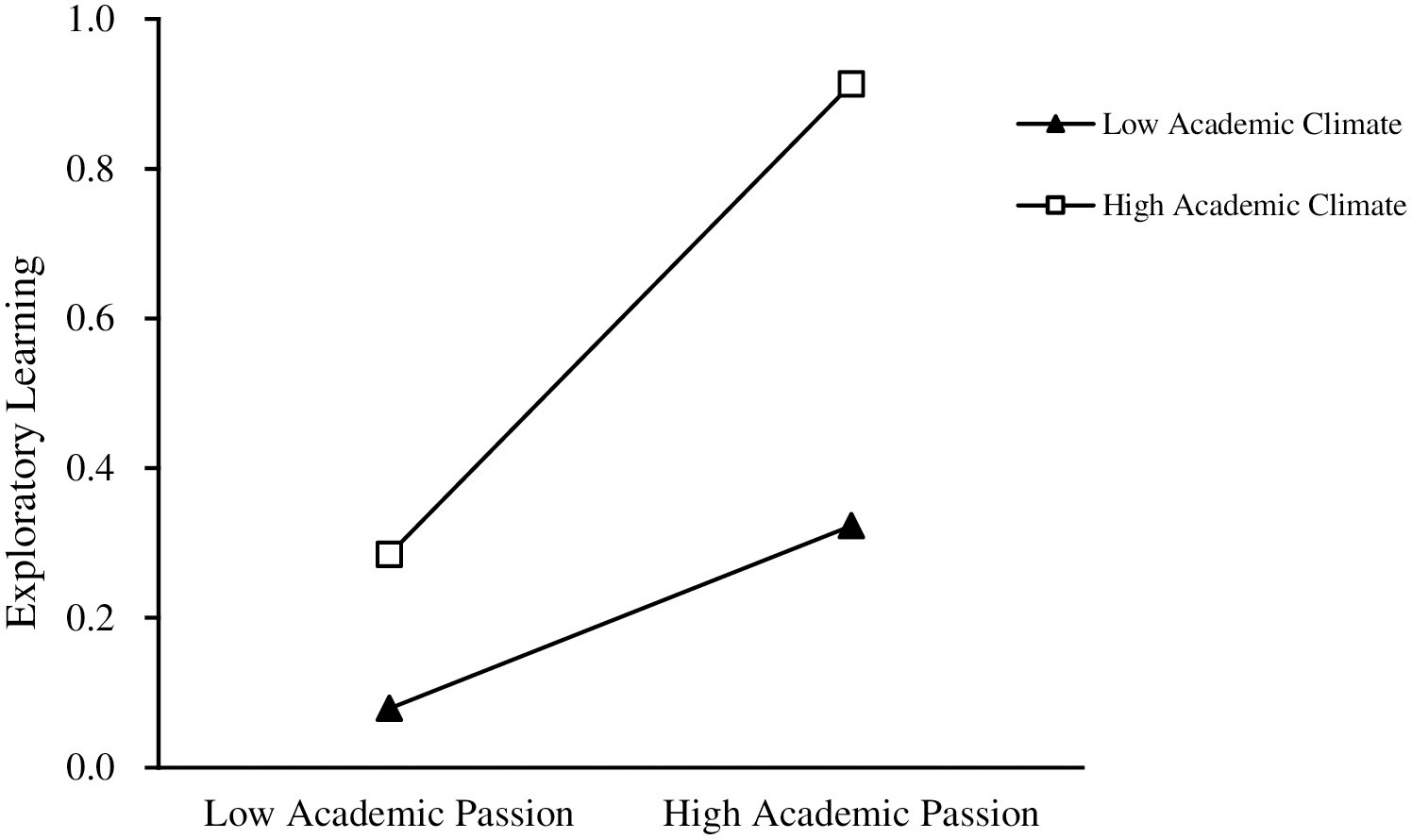
Moderating effect of academic climate on academic passion and exploratory learning.

**Fig 4:**
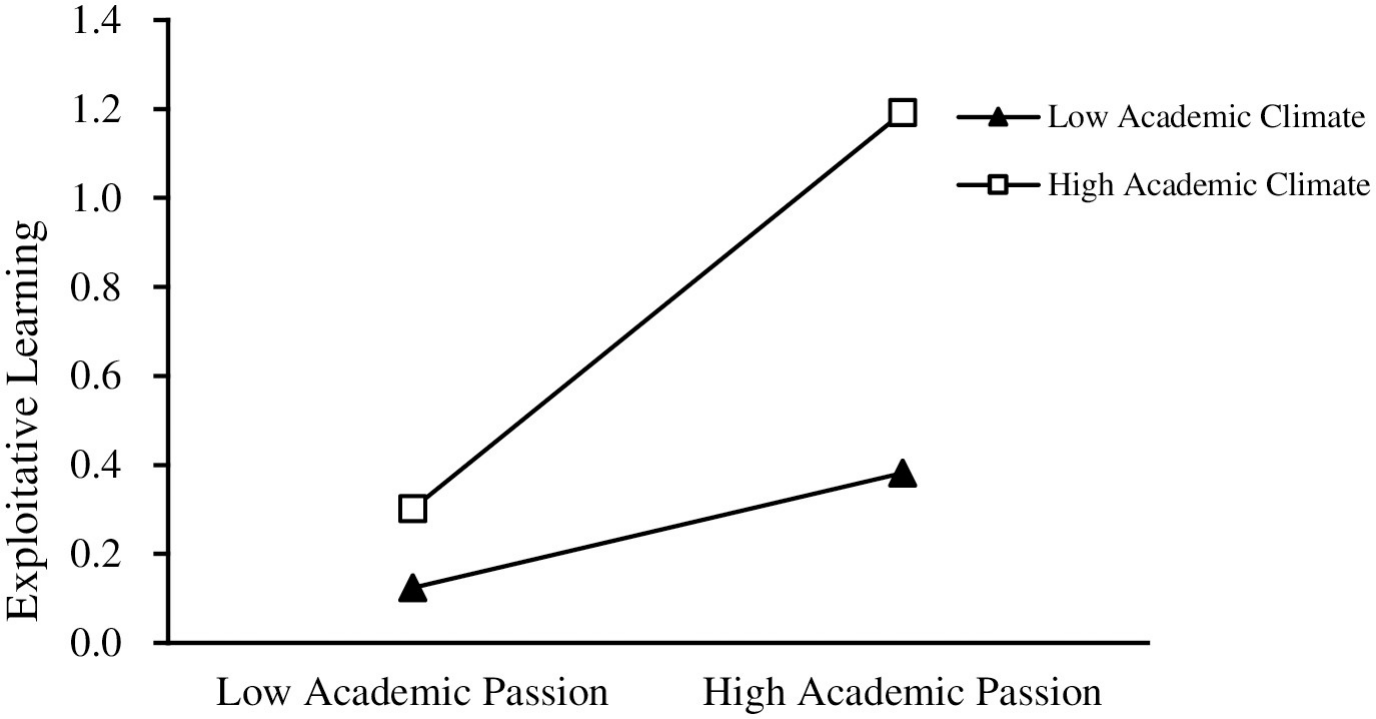
Moderating effect of academic climate on academic passion and exploitative learning.

To further test the moderating effect of academic climate between academic passion and ambidextrous learning (exploratory learning, exploitative learning). As shown in Figs 3 and 4, the academic climate positively moderates the direct relationship between academic passion and ambidextrous learning (exploratory learning, exploitative learning), and thus Hypothesis 4 and Hypothesis 5 are supported.

*Conditional process model test*. The "conditional process model" proposed by Hayes & Rockwood (2020) [72] was used to explore the mechanisms and boundary conditions of the independent variables on the dependent variable. To test the moderating effect of the academic climate on the "academic passion → ambidextrous learning → research engagement" pathway, a bootstrapping method of 10, 000 samples was also used. Table 4 shows the correlation coefficients, standard errors, and confidence intervals for the two paths of the conditional process model. It can be shown that there is a significant effect for both paths and therefore Hypothesis 6 and Hypothesis 7 are supported.

**Table 4:** Effect analysis for condition process model.

	AP→Exploratory Learning→RE				AP→Exploitative Learning→RE			
	Level	Estimate	S.E.	95%CI	Level	Estimate	S.E.	95%CI
AC	Low	0.018	0.016	[-0.007, 0.055]	Low	0.015	0.013	[0.062, 0.159]
	High	0.073	0.025	[0.032, 0.130]	High	0.104	0.025	[-0.005, 0.044]
	Odds	0.054	0.023	[0.015, 0.108]	Odds	0.089	0.023	[0.050, 0.142]

Note.AC = Academic climate; AP = Academic passion; RE = Research engagement.

## 5. Discussion

### 5.1. General findings

This study, grounded in Self-Determination Theory (SDT), explores the impact of academic passion on doctoral students’ research engagement, as well as the mechanisms of ambidextrous learning and academic climate. The findings reveal that academic passion is not only a profound emotional inclination towards research activities but more importantly, it serves to ignite intrinsic motivation, driving doctoral students to engage more actively in research. This is particularly significant from the perspective of SDT, which underscores the central role of intrinsic motivation in fostering positive individual behaviors. By introducing exploratory and exploitative learning as mediating variables, we further elucidate the mechanisms through which academic passion translates into concrete research engagement, unveiling the "black box" of this process. The main conclusions drawn from empirical analysis are as follows:

First, PhD students’ academic passion positively affects their level of research engagement. This finding aligns with SDT, which emphasizes the crucial role of autonomous motivation in spurring positive behaviors [5], with passion exerting a greater impact on certain positive outcome variables [73]. This implies that when doctoral students perceive academic research as part of their self-fulfillment, their engagement in research is heightened. This intrinsic drive encourages doctoral students to approach academic challenges with more vigor and willingness to invest time and effort.

Second, ambidextrous learning mediates the relationship between academic passion and doctoral students’ research engagement. This conclusion not only illustrates how academic passion is transformed into research engagement through these two distinct learning modes but also echoes findings that ambidextrous learning can mutually foster the effective enhancement of individual autonomous motivation and innovative performance [74, 75]. Exploratory learning encourages doctoral students to explore new knowledge and innovative methods, while exploitative learning aids them in making more effective use of existing knowledge. The combination of these two learning modes not only promotes the enhancement of doctoral students’ research capabilities but also strengthens their engagement in research activities.

Third, academic climate moderates the direct effects of academic passion on ambidextrous learning. Our data indicate that compared to a low academic climate, in a high academic climate environment, academic passion significantly strengthens its promotion of exploratory and exploitative learning among doctoral students. Academic passion brings about more positive emotions for doctoral students, and under the modulation of academic climate, these positive emotions are conducive to enhancing doctoral students’ self-efficacy in research, thereby generating two distinct types of learning behaviors: continually attempting to break knowledge boundaries and exploring, refining, and applying existing knowledge. This research conclusion aligns with findings that situational factors can have a significant impact on individuals’ behaviors [76].

Fourth, academic climate significantly enhances the positive impact of academic passion on doctoral students’ research engagement through ambidextrous learning. This not only highlights the environmental pathway critical to fostering doctoral students’ innovative capabilities [77] but also emphasizes academic climate as an important medium connecting doctoral students’ intrinsic motivation and behavioral performance. A vibrant academic atmosphere and abundant resources become fertile ground for nurturing innovative thinking [78], inspiring doctoral students to actively engage in academic exploration. In a rich academic climate, doctoral students with high academic passion tend to adopt a more proactive learning stance, collaboratively pursuing knowledge innovation with team members and deriving a sense of satisfaction from learning, thereby enhancing their research engagement. Conversely, in environments lacking a supportive academic climate, even doctoral students with academic passion may experience a reduction in motivation due to a lack of positive feedback, ultimately affecting the degree of research engagement.

### 5.2. Theoretical contributions

The following is a summary of this study’s theoretical contributions:

First, a new perspective illuminates the relationship between academic passion and PhD research engagement. Much of the previous research has emphasized the impact of academic passion on academic participation [79], academic performance [9], self-efficacy [80], satisfaction [26], and academic procrastination [81], among others, are under-explored in terms of research engagement. This study incorporated research investment as an outcome variable of academic passion in accordance with the self-determination theory and the analytical principle of "motivation-behavior-effect". On the one hand, it expands the outcome variables of academic passion and enriches the research literature on the positive effects of academic passion; On the other hand, it enriches the research on the impact of antecedent variables on research engagement, and helps to enrich the research scope and applicability of self-determination theory while providing new theoretical perspectives for research related to research engagement.

Second, the black-box mechanism between academic passion and research engagement has been uncovered. Existing studies examining the effect of academic passion on learning engagement are mainly based on social cognitive behavioral theory [82] and focus on subject category [83], learning environment [84], and type of passion [85], among other factors. However, these factors only relate to aspects such as individual background characteristics and the external environment, ignoring the effect of autonomous motivation on behavior. Considering that learning is the main activity of PhD students to improve their knowledge system, this paper focuses on the mediating effect of ambidextrous learning. The results suggest that ambidextrous learning mediates the positive impact of academic passion on PhD students’ research engagement. Thus, providing an alternative explanation for the process mechanism by which academic passion influences outcome variables. Opened the "black box" of the influence effect of academic passion and deepened the understanding of the mechanism of academic passion. The study in this paper also responds, in part, to the call for existing research to further explore the mechanisms of the role of academic passion from a self-determination theory perspective.

Third, by introducing an academic climate, the boundary effects of the role of academic passion are further clarified. Past studies have mostly examined the moderating effect of the effect of academic passion in terms of guide-student interactions [86], personal traits [9, 28], etc., and have lacked a focus on academic climate. It has been suggested that PhD students’ enthusiasm and interest in academic work can be unconsciously influenced by the academic climate [87]. A high academic climate is conducive to enhancing the academic passion of PhD students [16]. As a result, this study focuses on the moderating effect of academic climate, breaking through the limitations of previous studies that have used it only as an antecedent and mediating variable. Validated its moderating role between academic passion and exploratory learning, and between academic passion and exploitative learning.

Fourth, this study contrasts Self-Determination Theory with models such as Achievement Goal Theory and Social Cognitive Theory, further highlighting Self-Determination Theory’s unique contributions and advantages in explaining the relationship between doctoral students’ academic passion and research engagement. Achievement Goal Theory emphasizes the impact of types of academic goals on learning behavior and achievement [88], while Social Cognitive Theory underscores the central role of self-efficacy in learning and behavioral change [89]. This study finds that academic passion, as an intrinsic motivation, directly fosters research engagement, echoing the perspective in Achievement Goal Theory that mastery goals promote deep learning strategies [90]. Self-Determination Theory reveals the mediating role of ambidextrous learning and the moderating role of academic climate, constructing a comprehensive framework that clearly demonstrates how intrinsic motivation and external environment jointly promote positive academic behavior. Moreover, the moderating role of academic climate complements Social Cognitive Theory’s consideration of environmental factors, emphasizing the importance of a positive academic environment in stimulating academic passion and enhancing research engagement. This not only enriches the application of Self-Determination Theory but also provides significant theoretical and practical guidance for higher education practice.

### 5.3. Practical implications

Through specific recommendations and measures for doctoral students, universities, research institutions, and educational policies, this study aims to comprehensively enhance doctoral students’ academic passion, research engagement, and academic achievement. This, in turn, contributes to the improvement of higher education quality and innovation in academic research. Specifically:

Firstly, doctoral students themselves should recognize that academic passion is not only about personal love for academic research but also a key driver for research engagement and academic achievement. As an intrinsic motivator, passion guides a positive cycle of proactive behavior [54], providing a continuous source of energy to overcome challenges and difficulties encountered during research [91]. By revealing the crucial role academic passion plays in directly promoting research engagement, this study highlights the key impact of intrinsic motivation on the sustainability and efficiency of research activities. To fully utilize the potential of academic passion, doctoral students need to actively cultivate and protect their enthusiasm for academic pursuits. This means, beyond setting clear and challenging research goals, they should also actively integrate into the academic community, engage in deep exchanges and collaborations with mentors and peers, share research experiences, discuss academic issues, and participate in academic discussions to continually inspire and maintain academic passion. Doctoral students should also learn to effectively manage personal emotions and stress. Engaging in research discussions, academic conferences, and workshops not only broadens their perspectives but also provides more support and encouragement on the research journey, thus continuously enhancing the quality and efficiency of research engagement. Additionally, doctoral students should recognize that cultivating and maintaining academic passion is an ongoing process that requires continuous self-reflection and growth. Regularly assessing one’s research interests and goals, exploring new research methods and fields, and establishing connections with a broader academic circle are effective ways to cultivate academic passion. Through these methods, doctoral students can not only improve their research capabilities and academic output but also find more joy and fulfillment in their academic journey, achieving both personal career development and academic aspirations.

Secondly, universities and research institutions need to recognize the importance of creating a positive and healthy academic atmosphere in stimulating doctoral students’ academic passion and promoting their research engagement. This study finds that an optimized academic environment significantly enhances the positive impact of academic passion on ambidextrous learning, thereby further deepening research engagement. To achieve this goal, higher education institutions should commit to providing ample academic resources, including but not limited to access to the latest research literature, advanced research tools and equipment, and ample research funding support. These resources not only form the foundation for smooth research activities but are also key conditions for encouraging doctoral students to explore the unknown and create new knowledge. Additionally, by regularly organizing diverse academic activities such as lectures, seminars, and workshops, universities not only provide doctoral students with platforms to acquire new knowledge and skills but also promote exchange and cooperation within the academic community, thereby enhancing doctoral students’ sense of academic belonging and achievement. Moreover, creating opportunities for doctoral students to participate in a wider range of research projects and team collaborations not only helps improve their research skills and teamwork abilities but also enhances their love for and commitment to research through practical experience.

Finally, to fully realize the role of academic passion in doctoral students’ research engagement, doctoral training policies and educational practices urgently need to adopt more flexible and innovative strategies, especially in supporting ambidextrous learning paths. This study emphasizes that by encouraging both exploratory and exploitative learning paths, doctoral students’ research capabilities and participation can be effectively enhanced. Based on this, policymakers and higher education administrators should regularly organize workshops and seminars themed on ambidextrous learning, inviting experienced scholars to discuss practical strategies and methods for effectively integrating exploratory and exploitative learning in the research process. Not only does this provide doctoral students with opportunities to apply theory to practice, but it also inspires their enthusiasm for learning and innovative thinking. Additionally, mentors, as the "guides" on doctoral students’ academic paths and significant shapers of the learning environment [92], play an indispensable role in fostering ambidextrous learning strategies. Higher education institutions should strengthen support and training for mentors, encouraging them to adopt flexible and diverse guidance methods to meet the diverse research projects and learning needs of doctoral students. Mentors should guide doctoral students on how to integrate exploratory and exploitative learning in their research activities, thereby promoting their comprehensive enhancement of research capabilities and innovation in outcomes. Simultaneously, higher education institutions should reconsider and optimize evaluation and incentive mechanisms to ensure that the outcomes of doctoral students’ ambidextrous learning are fully recognized. By assessing doctoral students’ contributions to the discipline and the quality and depth of their ambidextrous learning outcomes, they can be better motivated to explore and grow on their research journey. In summary, through the implementation of these comprehensive strategies, the goal is to establish a healthy academic ecosystem that encourages both knowledge exploration and application, fosters innovation and collaboration, and provides solid support for doctoral students’ academic pursuits and career development.

### 5.4. Limitations and future research

While this study employed a multi-timepoint data collection method to reduce common method bias, its reliance on self-reported data from doctoral students may still be subject to some degree of common source bias. To further validate the findings of this study, future research could consider adopting experimental or quasi-experimental designs, or incorporating objective data sources, to enhance the accuracy and reliability of the results. Additionally, this research primarily explores the impact of academic passion on research engagement from the perspective of doctoral students, without fully considering the potential roles of external factors such as schools and supervisors. Factors such as the institution’s training policies and the supervisor’s support attitude may also influence doctoral students’ research engagement. Therefore, future studies could attempt to construct a more comprehensive theoretical model to examine how these external factors interact with doctoral students’ intrinsic motivations, jointly influencing research engagement. Lastly, given that the sample of this study is limited to doctoral students in China, the cultural universality of its conclusions remains to be further verified. The mechanism by which academic passion affects research engagement may vary across different cultural backgrounds. Hence, subsequent research should consider cross-cultural comparisons to explore and verify the universality and specificity of the findings of this study.

### 5.5. Conclusion

This study delves into the influence of academic passion on doctoral students’ research engagement based on Self-Determination Theory (SDT), along with the roles of ambidextrous learning and academic climate in this process. Our findings underscore the significant positive impact of doctoral students’ academic passion on their research engagement. Moreover, ambidextrous learning—comprising exploratory and exploitative learning—acts as a mediator, indicating that doctoral students can enhance their research engagement effectively through adopting diverse learning strategies. The moderating role of academic climate further highlights the criticality of a positive environment in stimulating academic passion and facilitating research engagement. These conclusions not only enrich the application of SDT but also offer guidance for higher education practices, particularly emphasizing the necessity of fostering a conducive academic atmosphere and supporting dual learning strategies. For policymakers in education, this study advocates the importance of creating supportive learning environments and optimizes doctoral students’ learning and research experiences by strategically encouraging dual learning paths. For higher education administrators, this study emphasizes the importance of constructing and maintaining a positive academic atmosphere, which aids in igniting doctoral students’ academic passion as well as their research engagement and innovative outputs. Future research directions include exploring the impact of academic climate across different disciplines and cultural contexts, and how educational policies and management practices can more effectively promote the application of ambidextrous learning strategies, further deepening our understanding of the motivational transformation mechanisms in the process of doctoral students’ research engagement.

## Supporting information

S1 File Supporting information. (DOCX)

S2 File Underlying data. (XLSX)
