# Supplementary material for: A study on the influence of academic passion on PhD students’ research engagement—The role of ambidextrous learning and academic climate
Source: PLoS One. 2024 Jun 3;19(6):e0303275. doi: 10.1371/journal.pone.0303275 (PMC11146691; doi:10.1371/journal.pone.0303275)
Supplement: S1 File — (DOCX) [file pone.0303275.s001.docx]

# Appendix

***Academic passion（AP）***

1: Being in academia makes me feel fulfilled

2: The experience of discovering new things makes me enjoy academic research even more

3: Academic research provides me with new and exciting experiences

4: I believe I have the qualities for academic research

5: No matter how difficult academic research is, I am always up for a challenge

6: Academic research does not interfere with my daily life (marriage and family, fitness and leisure, etc.)

7: Academics is the only thing that continues to excite me

8: Can't imagine my life without academic research

9：My emotional well-being often depends on the progress of my academic activities

10: If I could, I would devote all my time to academic research.

***Research engagement（RE）***

11: I'm happy to get up in the morning and do academic research

12: I feel energetic when conducting academic research

13: I can persevere even when research doesn't go well

14: I can recover quickly from mental fatigue when conducting academic research.

15: I am able to continue my academic research for a long time without taking breaks in between

16: I am strong and motivated when conducting academic research

17: Academic research inspires me

18: I am passionate about academic research

19: Academic research is challenging for me

20: I am proud of the achievements of my academic research

21: My academic research is purposeful and meaningful

22: I forget everything around me when it comes to academic research

23: When I conduct academic research, I have only research in mind.

24:I feel like time flies when I'm doing academic research

25: I'm happy when I'm fully engaged in my research

26: When doing academic research, it is difficult for me to stop

27: I immersed myself in academic research

***Exploratory learning***

28: I learn to acquire new knowledge and conduct new research

29: I tend to use new methods to solve problems

30: I often search for solutions to problems in unfamiliar areas

31: I focus my learning on knowledge that is new or far beyond the scope of my current studies

32: In learning, I tend to get a high reward, even if the risk is high

***Exploitative learning***

33: I study to improve the efficiency and quality of my current research

34: I focus my studies on knowledge closely related to the research at hand

35: I always look for solutions to problems in areas that I know well

36: I often use proven, generally accepted solutions to problems

37: In my studies, I tend to avoid risk and get more certain rewards

***Academic climate（AC）***

38: My school and team have a wealth of academic activities.

39: My school and team have a good academic ethos

40:My school and team have adequate academic resources

41: My school and team have open lines of academic communication

42: My school and team have a sense of co-operation and team spirit

43: My school and team have a good system of guidance and incentives for academic innovation
